# Effects of *houttuynia cordata* thunb. on rhinosinusitis by nasal irrigation: A protocol for systematic review and meta-analysis

**DOI:** 10.1097/MD.0000000000023634

**Published:** 2020-12-18

**Authors:** Dandan Wang, Tian Tian, Chao Liao, Ting Liu, Guangjun Tang, Li Tian

**Affiliations:** a Hospital of Chengdu University of Traditional Chinese Medicine; b Chengdu University of Traditional Chinese Medicine, Jinniu District, Chengdu, Sichuan Province, PR China.

**Keywords:** clinical trial, *houttuynia cordata* Thunb, nasal irrigation, protocol, rhinosinusitis, systematic review

## Abstract

**Background:**

The herba *Houttuynia cordata* Thunb. (HC) has anti-inflammatory, antibacterial and antiviral effects. Through nasal irrigation, the related preparation of HC is beneficial for treating rhinosinusitis or promoting recovery after an endoscopic sinus surgery. However, it remains controversial whether nasal irrigation with HC preparation can provide evidence-based clinical benefits for rhinosinusitis patients.

**Methods:**

With reference to the Preferred Reporting Items for Systematic Reviews and Meta-Analyses (PRISMA), 8 databases are perused to perform a methodical investigation of nasal irrigation with HC preparation and health-related results amongst rhinosinusitis patients. The main research purpose is to determine the influence of PRISMA standards on medical results pertaining to rhinosinusitis patients, including quantitative symptom recording and effective rate. With reference to the Cochrane Handbook, quality assessment of qualified papers is conducted using the Cochrane Risk Assessment Tool.

**Results:**

The results will be publicised through a peer-reviewed journal publication.

**Conclusion:**

The results of the systematic review will summarise evidences for the efficacy of nasal irrigation with HC preparation in rhinosinusitis treatment.

**Ethics and dissemination:**

Since this study involves a methodical investigation of issued medical papers, ethical authorisation and informed patient consent are not necessary.

## 1 Introduction

A common disease in otorhinolaryngology, rhinosinusitis is an inflammation of the sinus mucosa caused by viruses, bacteria or fungi. The incidence rate of rhinosinusitis amongst Chinese adults is as high as 8%.^[1]^ Rhinosinusitis causes apparent physical symptoms, including hyposmia, rhinorrhoea, nasal congestion and headache. The persistence of these symptoms can damage patients’ health, vitality and social function, affect their quality of life and work,^[2,3]^ and also cause depression and anxiety, thus impacting their psychological health.^[4]^

Since the paranasal sinuses are located in the skull and are connected with the nasal cavity through the opening, intranasal administration can effectively target the local inflammatory process by the nose and paranasal sinuses, concentrate the active ingredients, and attain a rapid effect. Studies have determined that intranasal corticosteroids and nasal saline irrigation are considered safe and effective as first-line treatment for rhinosinusitis.^[5]^ Thus, intranasal administration is deemed as the best treatment for rhinosinusitis. Low-volume devices such as drops and nebulizers have a limited utility in rhinosinusitis treatment due to their limited extension into the sinus.^[6]^ Meanwhile, nasal irrigation is a typical, low-cost and well-tolerated method for rhinosinusitis and post-endoscopic sinus surgery; it can directly remove local inflammatory mediators and antigens,^[7]^ reduce postoperative scab, remove inflammatory mucus, improve mucociliary function and reduce mucosal swelling.^[6]^ As a mechanical intervention, nasal saline irrigation can clear retained viscous secretions. However, it cannot directly enhance mucosal immunity and relieve congestion, and can only temporarily replace the damaged mucociliary clearance.^[6,8]^ As an external therapy in traditional Chinese medicine, nasal herbal irrigation not only cleans the nasal cavity, but also has a therapeutic effect comparable to drugs. For several years, it has been developed in the aspect of treatment of nasal diseases.

Known as “Chinese medicine antibiotic”, *Houttuynia cordata* Thunb. (HC) is a medicinal and edible Chinese medicine with a strong anti-inflammatory effect. Studies have found that HC can reduce nasal mucosal congestion and swelling and sinus ostium obstruction,^[9]^ control focal infection of pathogenic bacteria, effectively repair pathological changes of nasal mucosa in rat models of rhinitis, and exert anti-inflammatory activity.^[10]^ Due to the anti-inflammatory,^[11]^ anti-bacterial,^[12]^ anti-viral^[13]^ and mucosal immune regulation of HC,^[14]^ its association with nasal irrigation has great potential benefits for rhinosinusitis treatment. Studies have indicated that flushing the surgical cavity with HC after an endoscopic sinus surgery (ESS) can reduce mucosal inflammation and improve postoperative symptoms.^[15,16]^

Although multiple studies have provided evidence on the beneficial effects of nasal irrigation with HC in rhinosinusitis treatment, it remains controversial whether this method can provide evidence-based clinical benefits for rhinosinusitis patients after ESS. Moreover, the method's effectiveness and safety have not been systematically reviewed. As such, higher-quality evidences are required to draw reliable conclusions regarding this therapy. Relatively, this study conducts a systematic review and meta-analysis of randomised controlled trials (RCTs) of nasal irrigation with HC in the treatment of rhinosinusitis or postoperative patients. In this case, it is necessary to include published RCTs and conduct an evidence-based research.

## 2 Methods and design

This study had been registered at Open Science Framework (OSF) (https://osf.io/dfa3x). The registration number is Doi: 10.17605/OSF.IO/DFA3X.

### 2.1 Search strategy

A literature search on studies through 23 October 2020 was performed on the Cochrane Library, PubMed, the Wanfang Chinese Digital Periodical and Conference Database, Embase (Excerpta Medical Database), the VIP Chinese Science and Technique Journals Database, the China National Knowledge Infrastructure (CNKI) database, and the Chinese Cochrane Centre's Controlled Trials Register platform. Other sources were the Chinese Clinical Trial Registry Center and the references of the included studies.

Eligible studies will be selected according to the inclusion criteria. Should there be insufficient data, the original authors of the respective study will be in touch with to acquire the remaining necessary data. Table 1 comprehensively illustrates PubMed's search technique. For other databases and sources, comparable approaches will be implemented.

**Table 1:** Search strategy for PubMed.

Number	Search terms
#1	houttuynia^∗^
#2	sinus infection^∗^
#3	^∗^sinusitis
#4	#2-#3/OR
#5	irrigation
#6	washing
#7	douching
#8	lavage
#9	rinsing
#10	#5-#9/OR
#11	#1 AND #4 AND #10

### 2.2 Inclusion and exclusion criteria

#### 2.2.1 Inclusion criteria

##### 2.2.1.1 Studies

Only prospective RCTs of nasal irrigation with HC solution for rhinosinusitis treatment will be included. Eligible languages will be limited to either Chinese or English. These criteria will be expanded should less than five qualified RCTs will be acquired from the methodological investigation.

##### 2.2.1.2 Participants

All patients diagnosed with sinusitis, or rhinosinusitis, or postoperative sinusitis will be included.

##### 2.2.1.3 Interventions

Only those papers with interventions involving nasal irrigation and HC against standard placebo or medicinal procedures will be included. For control groups against experimental ones, the established interventions are the following:

(1) HC vs conventional drug;
(2) HC combined with conventional drug vs conventional drug only;
(3) HC combined with other complementary therapies vs other complementary therapies only; and,
(4) HC vs placebo or no therapy.

##### 2.2.1.4 Outcomes

The primary pre-specified outcomes will include the clinical effectiveness and quantitative scoring of certain symptoms, including nasal obstruction, nasal discharge, facial pain and hyposmia. Quantitative evaluation of local examinations such as nasal endoscopy or CT scan will be incorporated as secondary outcomes. The aforementioned results will be regulated upon review depending on the determined results in the qualified papers. If these results will be used in the system review, special care will be undertaken to avoid selective reporting bias.

#### 2.2.2 Exclusion criteria

The exclusion criteria are the following:

(1) Patients with nasal tumour, fungal sinusitis or odontogenic maxillary sinus infection;
(2) Duplicate or incomplete data that cannot be obtained after contacting the original authors; and,
(3) Repeatedly published studies.

### 2.3 Data abstraction

All articles obtained from the aforementioned search strategy were imported into Endnotes X9 to eliminate duplicate studies. The articles’ abstracts were screened by two independent investigators with reference to the inclusion and exclusion criteria; afterward, the screened full text was retrospectively analysed. Finally, the included articles were organised in a Microsoft Excel file.

**Figure 1:**
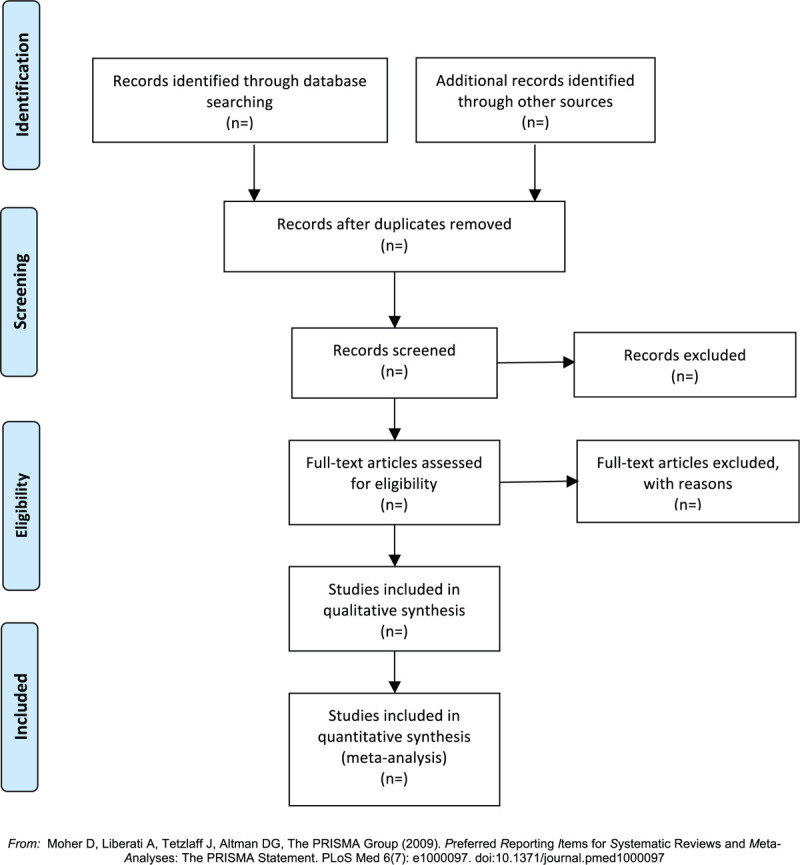
Flow diagram of study selection.

Raw data from the included articles will be independently extracted by two authors and will include study region, author information, publication, follow-up information, patients’ ages, diagnosis, sample size, outcome measures, intervention and control methods, randomisation method, blinding, incomplete outcome data, withdrawal, and dropout and adverse events. The dosage form of HC will also be extracted. The extracted data will be verified for accuracy and completeness by a third investigator. The outcome variables will be obtained for all included studies. Any conflict will be settled through discussion or arbitration by third-party reviewers. Basing from the search technique and eligibility evaluation, Figure 1 illustrates the produced Preferred Reporting items for Systematic Reviews and Meta-Analyses (PRISMA) diagram to depict the studies’ flow.

### 2.4 Quality assessment

The methodological quality of the included RCTs will be evaluated by three investigators using the Review Manager (RevMan) software version 5.3, in accordance with the Cochrane Handbook for Systematic Interventions.

Included studies will be evaluated based on the following criteria: allocation concealment, selective outcome reporting (SOR), other risks of biases, incomplete outcome data, randomisation generation, and blinding of study personnel/patients and outcome assessors.^[17]^

Trials sponsored by a relevant manufacturer, in which baseline characteristics are not alike amongst intervention groups, will be identified as a bias. If at least ten trials have reported the primary outcomes, publication bias will be evaluated by funnel plots.

### 2.5 Statistical analysis

RevMan 5.3 will be used to summarise data for meta-analysis. The relative risks, along with 95% confidence intervals (CIs), will express the measurements of dichotomous data. Meanwhile, continuous data will be expressed as mean differences along with 95% Cis; *P* < .05 will be identified as statistically significant. Interstudy heterogeneity will be assessed by I^2^. *P* ≤ .1 and I^2^ > 50% represent a considerable heterogeneity, and the random effect model and subgroup analysis will be applied. When *P* ≥ .1 and I^2^ < 50%, data heterogeneity is low, hence the fixed effect model will be applied for merging.

Then, when the dosage form of HC in certain studies is similar, the respective trials will be summarised. From these similarities, specific subgroups will be examined. If the P-value test for heterogeneity after subgroup analysis is less than 0.1, a sensitivity analysis will be performed to assess the robustness of the results. Meta-analysis will be conducted again after elimination of low-quality studies. Moreover, the influence of statistical models on the outcomes will be evaluated. In this case, statistical significance will be attained if *P* < .05.

### 2.6 Grading of recommendation assessment, development and evaluation (GRADE) quality assessment

Implementing the Grading of Recommendation Assessment, Development and Evaluation (GRADE) approach, two impartial evaluators will determine the level of confidence of the results. In case of conflicts, they will be typically settled by means of reconciliations; should the conflict persists, a third evaluator will be conferred to come up with the final verdict.

## 3 Discussion

Nasal saline irrigation is one of the listed treatments in the clinical guidelines for sinusitis^[18]^; it can clean the nasal cavity and protect the mucosa. Alongside an HC solution, it can exert antibacterial, anti-inflammatory and antiviral effects on the lesions in rhinosinusitis treatment and postoperative recovery. RCTs have demonstrated that this combined therapy is superior to saline irrigation,^[19]^ but the guidelines do not recommend it. Perhaps, the underlying reason is the inadequacy of high-quality medical efficiency or procedural researches.

Pharmacological studies have found that HC can play an anti-inflammatory function by reducing inflammatory cytokines and chemokines levels.^[20]^ HC's active components also have antibacterial and antifungal activities,^[21]^ and are often used for treating infectious diseases. Nasal irrigation with HC solution after ESS can clean the nasal cavity, reduce scab formation, promote discharge of secretions, and eliminate mucosal inflammation and oedema. It can also shorten cleaning times of the surgical cavity, reduce occurrences of mucosal lesions like vesicle granulation, and promote the epithelisation of the surgical cavity to enhance cure rates.^[22]^ Its efficacy and safety have been explored in several clinical studies. However, there is no comprehensive analysis and evaluation based on these studies to reduce biases and reach a scientific conclusion on the efficacy and safety of nasal irrigation with HC solution. Therefore, it is of utmost significance in this systematic review to determine nasal irrigation with HC solution as an alternative therapy for rhinosinusitis patients, especially after ESS.

Presently, there are several clinical studies concerning the beneficial effects of nasal irrigation with HC on rhinosinusitis and postoperative recovery, but evidences remain limited. In the future, high-quality and large-sample RCTs in this aspect are required to enhance the acceptability of this mode of therapy. Moreover, this methodical investigation is deemed to have some possible limitations. For one, this research emphasises on the heterogeneity of the empirical outcomes and the inferior quality of the current articles. Therefore, descriptive methods will be necessarily used to present the findings. This study protocol is designed based on the research data or results in existing published (and unpublished) literature, and in accordance with nasal irrigation and HC for treating rhinosinusitis. It is desired that the application of this approach will aid in generating comments and positive criticism regarding the intended research procedure prior to commencing the research proper.

To conclude, this planned methodical investigation will serve as a foundation for determining the medical influence of nasal irrigation with HC amongst rhinosinusitis and postoperative patients, whether in China or abroad. Furthermore, it will aid in underscoring the matters that necessitate a more thorough design in this research aspect.

## Author contributions

**Conceptualization:** Dandan Wang, Tian Tian, Li Tian.

**Data curation:** Tian Tian, Chao Liao, Guangjun Tang.

**Funding acquisition:** Li Tian.

**Investigation:** Tian Tian, Chao Liao, Guangjun Tang.

**Methodology:** Dandan Wang, Guangjun Tang.

**Software:** Chao Liao, Guangjun Tang.

**Supervision:** Ting Liu, Li Tian.

**Visualization:** Chao Liao.

**Writing – original draft:** Dandan Wang.

**Writing – review & editing:** Dandan Wang, Tian Tian, Li Tian.
